# VarB: a variation browsing and analysis tool for variants derived from next-generation sequencing data

**DOI:** 10.1093/bioinformatics/bts557

**Published:** 2012-09-13

**Authors:** Mark D. Preston, Magnus Manske, Neil Horner, Samuel Assefa, Susana Campino, Sarah Auburn, Issaka Zongo, Jean-Bosco Ouedraogo, Francois Nosten, Tim Anderson, Taane G. Clark

**Affiliations:** ^1^Faculties of Epidemiology & Population Health and Infectious & Tropical Diseases, London School of Hygiene and Tropical Medicine, London WC1E 7HT, ^2^Malaria Programme, Wellcome Trust Sanger Institute (WTSI), Cambridge CB10 1SA, ^3^Faculty of Health, Cranfield University, Cranfield MK43 0AL, UK, ^4^Institut de Recherche en Sciences de la Sant, Bobo-Dioulasso, Burkina Faso, ^5^Shoklo Malaria & Mahidol-Oxford Clinical Research Units, Mae Sot, Tak, Thailand and ^6^Texas Biomedical Research Institute, San Antonio, TX 78245, USA

## Abstract

**Summary:** There is an immediate need for tools to both analyse and visualize in real-time single-nucleotide polymorphisms, insertions and deletions, and other structural variants from new sequence file formats. We have developed VarB software that can be used to visualize variant call format files in real time, as well as identify regions under balancing selection and informative markers to differentiate user-defined groups (e.g. populations). We demonstrate its utility using sequence data from 50 *Plasmodium falciparum* isolates comprising two different continents and confirm known signals from genomic regions that contain important antigenic and anti-malarial drug-resistance genes.

**Availability and implementation:** The C++-based software *VarB* and user manual are available from www.pathogenseq.org/varb.

**Contact:** taane.clark@lshtm.ac.uk

## 1 INTRODUCTION

Massively parallel sequencing (MPS) technologies are providing whole-genome data on organisms that cause or have disease. These data are being used to catalogue genomic diversity, and in the context of research in humans, inform genome-wide and fine-scale mapping projects. The technologies are capable of sequencing a small number of human genomes in a single run, making high throughput of human genomes technically possible. Pathogen genomes are much smaller, making them tractable for large genome diversity studies, enabling the tracking of their evolution over time and space. They are also more amenable for whole-genome association studies, leading to the identification of variants associated with phenotypes such as drug resistance. To take full advantage of genetic variation exposed by whole-genome sequencing across pathogen (and other) organisms, the development of interactive analysis and real-time visualization tools is essential.

The variant call format (VCF) has become the recognized data type for listing genomic variants, including single-nucleotide polymorphisms (SNPs) and insertions and deletions (indels), usually derived from processing alignment files. The VCF format was developed by the 1000 Genomes project and has been adopted by large-scale genome projects [e.g. UK10K, dbSNP (Danecek *et al.*, 2011)]. It facilitates multi-sample curation and identification of SNPs, short indels and other types of structural variants and sample meta-data. The software suite VCFtools implement utilities for processing files (Danecek *et al.*, 2011). Software to visualize VCF files is available (Carver *et al.*, 2012; Fiume *et al.*, 2010; Thorvaldsdttir *et al.* 2012), but they perform limited or no population and statistical genetic analyses in real time directly from multi-sample files. New tools are urgently required because of the increasing use of MPS technologies in genomic epidemiological studies, and the need to rapidly translate the sequence variation into further experiments. This need has motivated the development of an all-in-one variant browsing and analysis software—*VarB*.

## 2 FEATURES OF VarB

*VarB* is a standalone C++ software tool, which visualizes (un)phased polymorphisms in a VCF file by sample, genetic region and quality. The basic inputs are a reference genome (*fasta*), variant (VCF) and annotation (*gff*) files. Complete genomes or user-specified regions (e.g. chromosome) may be inputted and viewed, with variant genotypes being colour coded. The variants displayed and their number will change depending on the quality and read depth filtering selected, allowing users to assess the robustness of the data and analysis. Sequence data with genes, exons, coding regions and strand-dependent codons are marked at appropriate zoom levels, and tracks summarizing GC content, relative variant density and results from data analysis are presented. The Tajimas *D* metric (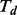) (Tajima, 1989) is implemented and is a method for distinguishing between a DNA sequence evolving randomly (‘neutrally’, values close to 0) and one evolving under a non-random process, including directional selection (low negative values) or balancing selection (high positive values). The population differentiation *Fst* measure is also implemented (Weir, 1996) and quantifies allele frequency differences between user-specified populations, with values ranging between 0 (no difference) and 1 (complete differentiation). It is possible to export the graphics and analysis outputs. *VarB* was developed using the Qt cross-platform and user interface framework (qt-project.org).

## 3 APPLICATION TO *PLASMODIUM FALCIPARUM* DATA

**Fig. 1.**
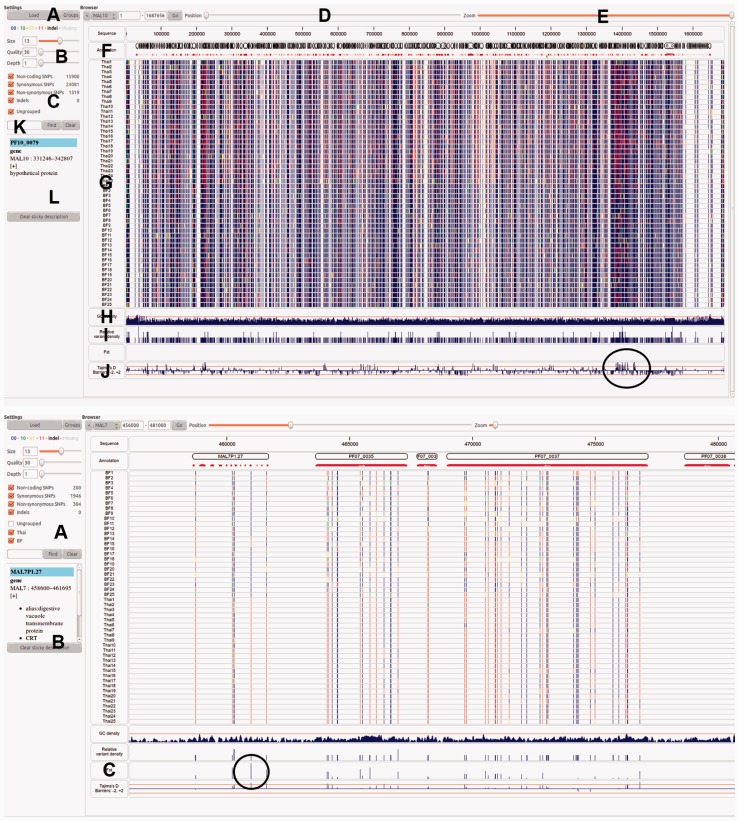
(Top) Chromosome 10 (MAL10): (**A**) loading of files (*fasta*, *gff*, *VCF*) and defining groups for *Fst* analysis; (**B**) colour coding of genotypes and alleles, and the setting of minimum quality and read depth; (**C**) variant and group inclusion for display; (**D**) position slider; (**E**) zoom slider; (**F**) annotation window; (**G**) display window where each row represents a different sample (BF1-25, Thai1-25) and variants colour coded (see B); (**H**) GC content track; (**I**) variant density; (**J**) Tajimas *D* track, with a region of high values circled (including the PF_10_0355 gene); (**K**) gene search tool; (**L**) gene information. (Bottom) Region of MAL7 (456 k–481 k): (**A**) inclusion of BF and Thai groups for analysis; (**B**) BF25 isolate information at position 461047 in the MAL7.1.27 gene, including a genotype call of 0/0 (reference allele); (**C**) *Fst* values for each SNP, with the highest value (0.92, circled, see B).

We demonstrate the functionality of *VarB* using *P. falciparum* (Pf) whole-genome (14 chromosomes, 23 Mb, ~81% AT content) sequence data from Burkina Faso (BF, *n *= 25) and Thailand (*n *= 25). The raw data are from a Pf genomic diversity study (Manske *et al.*, 2012; SRA Study ERP000190). The Illumina 54/76-base paired reads were mapped to the 3D7 reference genome (v3.0) using *smalt* (www.sanger.ac.uk/smalt) and processed as described previously (Robinson *et al.*, 2011) to construct VCF (v4.1) files consisting of SNPs and indels. Across the 50 samples, 46 283 SNPs (density 1 every 500 bp) were identified and summarized in a combined VCF file. We focus on those 23 942 (51.7%) SNPs with minor alleles observed at least twice. Figure 1 (top) shows the SNP data (*n *= 1790 loci, 7.5%) for chromosome 10. Estimation of 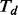 identified a large region (positioned ~1.4 Mb), potentially under balancing selection (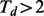). This region includes highly polymorphic antigenic-determining genes from the merozoite surface protein 3 family [*msp3* (PF10_0345); *msp3.8* (PF10_0355)], known to be under diversifying selection, and considered potential vaccine candidates (Ochola *et al.*, 2010). By specifying the two population groups (BF and Thailand), the population differentiation measure *Fst* was calculated. There were 1153 (4.8%) SNPs with near or complete population differentiation (*Fst* values > 0.95) across the genome. Loci involved in anti-malarial drug resistance often show high differentiation (Wootton *et al.*, 2002). Figure 1 (bottom) shows one region of interest in chromosome 7 containing the chloroquine resistance transporter (*PfCRT*, MAL7.1.27, maximum *Fst* 0.92). This locus is known to confer resistance to anti-malarial chloroquine-based drugs, with established differences in the haplotype structure between Southeast Asian and African isolates (Wootton *et al.*, 2002). Other polymorphisms known to confer sulfadoxine-pyrimethamine drug resistance, namely regions around *PfDHFR* and *PfDHPS* genes (Pearce *et al.*, 2009) had high *Fst* values (>0.80) (data not shown).

## 4 DISCUSSION

The translation of sequence variation into further laboratory experiments, treatments and point of care interventions, requires the ability to interrogate genomic data. *VarB* processes files of VCF format, displays the variants by position and quality as well as comparing them between groups to establish informative genetic markers and regions under selection. An advantage of the software is that it performs population and statistical analysis in real-time and does not require calculations to be performed elsewhere. We have presented data from 50 Pf isolates to demonstrate the utility of the software and highlighted regions with differing allele frequencies that coincide with known drug-resistance loci (e.g. *PfCRT*) as well as known vaccine candidates arising from considering regions under balancing selection (e.g. *msp3.8*). It is possible to process many hundreds of Pf or smaller genomes (e.g. bacterial) simultaneously. However, to aid processing of numerous much larger genomes, such as human, the software is capable of reading in single chromosomes. The modular computing architecture provides the flexibility to incorporate a number of extensions. These include the capacity to process BCF/BCF2 files, reading in informative meta tracks (e.g. genomic uniqueness), calculating other population genetic statistics and performing tests of association with a phenotype. Increased utility will also be possible through updates to the VCF format, to identify and annotate variants of greater size, such as large deletions.
